# Designing an evidence based community pharmacy service specification for a pharmacogenomic testing service

**DOI:** 10.1007/s11096-022-01483-8

**Published:** 2022-10-10

**Authors:** Tim Rendell, Julie Barnett, Sion Scott, David Wright

**Affiliations:** 1 University of Bath, Claverton Down, Bath, BA2 7AY UK; 2 University of Leicester, University Rd, Leicester, LE1 7RH UK

**Keywords:** Behaviour change techniques, Community pharmacy, Pharmacogenomics

## Abstract

**Background:**

Pharmacogenomics is a novel arena of medicine that uses patients’ Deoxyribonucleic Acid to support pharmacists and prescribers selecting the most appropriate medicine for patients.

**Aim:**

To review and validate a service specification for a pharmacogenomics testing service.

**Method:**

Consensus methods (Delphi method and the Nominal Group Technique) were deployed. A consensus panel comprising of pharmacists, prescribers and patients was convened to participate in the co-design process. Panel members were first surveyed to obtain their views on Behaviour Change Techniques identified as necessary for the service in a previous study. Following this, a workshop was convened to discuss, agree and confirm details for the service specification and recommend strategies for operationalisation. Outputs from the workshop were used to inform a final version of the service specification.

**Results:**

From the consensus panel (pharmacists (n = 6), general practitioners (n = 3) and patients (n = 3)), strategies for operationalisation of nine Behaviour Change Techniques were agreed as being required. In addition, several unique and innovative strategies for implementation of the community pharmacy service were identified and included in the service specification.

**Conclusion:**

The research shows that to encourage community pharmacist engagement in providing a pharmacogenomic testing service and prescriber acceptance of recommendations for any changes to patients’ prescriptions, a multi-professional launch event is required. To agree communication strategies and professional boundaries, training in clinical decision making and patient support materials are required as is guidance on how to deliver the service in a standardised manner. Finally, healthcare professionals would be reassured by the provision of an expert help-line for any complex patients.

## Impact statements

- The elements identified within the service specification can be used to inform the development of community pharmacy led PGx services.
- To optimise engagement with such services it is important for different stakeholders to agree professional boundaries and methods of communication, to include appropriate training in clinical decision making and to ensure that expert support is available when necessary.
- The service specification could be used as a baseline for a future trial to test effectiveness of PGx service implementation through community pharmacies.

## Introduction

Personalised medicine is the blended application of “*genetic, environmental, lifestyle, and other unique patient or disease characteristics to guide drug selection and dosage*” [1]. Pharmacogenomics (PGx) is a fast evolving branch of personalised medicine using DNA to predict an individual’s response to drugs with an outcome of better drug and safer dose selection, quicker and improved patient care and reduced costs for the healthcare system. By 2025, the new National Health Service (NHS) genomics medicine service could be incorporated into routine care [2]. Community pharmacists could be an integral part of this service, from administering the test, explaining test results to patients, to alerting prescribers to significant drug-gene interactions (DGI). To date, PGx testing services in community pharmacy have been implemented and evaluated in several countries including the USA [3], Canada [4] and the Netherlands [5]. Furthermore, translation of PGx testing results into actionable data in clinical practice is now well established and several international organisations have developed evidence-based guidance to recommend changes to prescribing where a drug–gene pair has been detected. The largest groups are the Clinical Pharmacogenetics Implementation Consortium (CPIC), the Dutch Pharmacogenetics Working Group (DPWG) and the Canadian Pharmacogenomics Network for Drug Safety (CPNDS). However, the exact interface with community pharmacists in the United Kingdom (UK) is so far unclear.

Following a scoping study, it has been identified that community pharmacy led PGx services have not yet been reported in the UK. The study identified the importance of patient interest, pharmacist engagement, training, supporting information for pharmacists and prescriber acceptance [6].

This paper reports on the final part of a three phase research study to co-design a PGx testing service. The first phase utilised three separate focus groups of pharmacists, prescribers and patients, and used reflexive thematic analysis [7]. The enablers for implementing a PGx testing service in community pharmacy were identified as: In-principle receptiveness and Appreciation of the benefits. The barriers to implementation were identified as: Lack of implementation resources, Ambiguity about implications for implementation and Inter-professional relationship challenges [8].

The second phase mapped data from the pharmacist focus group onto six domains of the Theoretical Domains Framework (TDF) [9]: Knowledge, Skills, Social/ professional role and identity, Optimism, Beliefs about Consequences, and Environmental context and resources. The Theory and Techniques Tool (TATT) [10] was subsequently used to identify the BCTs [11]. Finally, consensus methods (Delphi method and the Nominal Group Technique (NGT)) [12] were used to identify the most appropriate BCTs which were: Review outcome goal(s), Feedback on behaviour, Instruction on how to perform behaviour, Demonstration of the behaviour, Credible source and Adding objects to the environment [13].

This study reports the final phase which involves a mixed participant consensus panel being deployed to gain critical comment, and to determine the operationalisation of the identified BCTs from the second phase to develop a service specification for delivery of a community pharmacy based PGx service in England.

### Aim

To review and validate a service specification for a PGx testing service.

### Ethics approval

This study protocol gained approval from the NHS Health Research Authority (20/HRA/4147) on 03.09.20 for the prescriber group. For the pharmacist and patient groups ethical approval was given by The Research Ethics Approval Committee for Health (EP 19/20 069) on 11.11.20 at the University of Bath.

## Methods

A co-design panel was established and all members completed a survey followed by a virtual co-design workshop.

### Study panel members, sampling and recruitment

Pharmacists (n = 6), general practitioners (GP) (n = 3) and patients (n = 3) were included in a co-design panel purposively selected to represent a diverse range of gender, experience, roles, geography, setting and size of business. All the panel members had previously attended a focus group or consensus panel to maintain the integrity of the co-design methodology. Expressions of interest were sought through personal invitation based on panel member characteristics and their level of engagement in previous sessions.

### Data collection

#### Pre workshop survey

In preparation for the workshop, an online survey was developed which invited pharmacist and GP panel members to rate a range of options for operationalisation of the service. These were each linked to a BCT selected in the previous phase of this study. The survey questions and the majority of options were derived from the pharmacist consensus panel discussion whilst a few were developed by the research team. For the question “What should be included in a GP engagement pack to overcome any inter-professional barriers?”, respondents were invited to provide their ideas using a free text box as the options were not clear. The surveys were administered using the Momentive Inc® platform [14].

Panel members voted on those which were operationally appropriate in their personal experience by selecting one of four options: “must do”, “should do”, “could do” and “must not do”. Panel members were also asked to provide additional ideas or options for operationalisation of the BCT in a community pharmacy setting as free text. The survey was piloted to ensure clarity of understanding by three healthcare professionals and slightly amended following feedback for clarity (Table 1). Patients were not asked to contribute to the survey because it was asking the healthcare professionals to comment specifically on their roles and needs for service delivery and many of the options were professionally technical in nature. To assist panel members with completion of the survey, additional information was provided including an example PGx test report and a summary of the outcomes from the previous pharmacist consensus panel. The draft service specification was also provided by the researcher as a template for discussion.

**Table 1:** Pre workshop survey questions

Barrier/enabler	BCT	Survey question
In principle receptiveness	1.7 Review outcome goal(s)	To provide positive feedback to pharmacist to demonstrate effective service delivery, what should the outcome goal of the PGx testing service be?
Appreciation of the benefits	5.6 Information about emotional consequences	Pharmacists told us that patients’ may be worried if they find out that their medicines may have been inappropriate, and they need guidance on how to handle this. How should we address this?
	9.2 Pros and Cons	Pharmacists told us that they would like to know the pros and cons of the service to encourage them to take part. How could we best communicate these?
Lack of implementation resources	3.2 Social support (Practical)	How do we ensure the whole pharmacy team, peers and line managers are fully engaged prior to and during implementation?
Lack of guidance to design the service	12.5. Adding objects to the environment	What supporting materials should be produced?
Lack of knowledge for service delivery	4.1. Instruction on how to perform behaviour	What training is required to inform and demonstrate to community pharmacists how to offer and conduct a PGx test?
	6.1 Demonstration of the behaviour	
Lack of knowledge for clinical decision making	2.2. Feedback on behaviour	What format and frequency should regular summary updates and peer benchmarking information on testing outcomes be?
		What do community pharmacists (and GPs) need to do to ensure that they have the knowledge to make safe and appropriate clinical decisions based on the report provided?
Interprofessional relationship challenges	9.1 Credible source	What should be included in a GP engagement pack to overcome any inter-professional barriers?
		Who should identify the need for the patient to have the test after the prescribing of a new medicine to overcome any inter-professional barriers?

#### Workshop

The online consensus panel was convened using the Microsoft® Teams platform. The rated results for each strategy for operationalisation from the pre workshop survey were presented to all the panel members. At the consensus panel, each barrier and enabler was reviewed and the respective strategy options were either selected, merged or deselected, with the survey results helping to inform panel members contributions. All three participant groups commented upon and shared their opinions on the appropriateness, applicability and practicality for each proposed strategy to support the enablers and address the barriers from their individual professional or lay perspective.

The discussion of the online consensus panel was transcribed by an external company, Transcript Divas Limited® [15] into an anonymised orthographic transcript. It was then imported into the data storage and analysis software Taguette® [16]. Finally, the different strategies were ascribed to the relevant healthcare professional(s) and placed in chronological order of service delivery to create a service specification. Quotations were selected for presentation in the results based on their impact to support the discussion.

### Data analysis

The pre workshop surveys were analysed and reported as a % score with the following calculation: “must do” (3 points), “should do” (2 points), “could do” (1 point) and “must not do” (− 1 point). The results were reported as a % of the maximum number of 27 potential points: (*participants *(*9*)* X “must do” *(3) = 27). Those options achieving an average weighting of 65% or above were included for discussion at the consensus panel [17].

Following the consensus panel, the verbatim transcript was analysed, and at each point of consensus the recommended strategy options and comments for operationalisation were extracted and included in the final version of the service specification.

## Results

### Pre workshop survey

Options for inclusion in the service specification were rated by all pharmacist and GP panel members (n = 9) using the online survey and are summarised in Table 2. The option that most panel members selected as “must do” was written guidance/ Standard Operating Procedures (SOPs) for pharmacists on how to offer and perform the test whilst the option that most panels selected “must not do” was using the number of tests completed as the outcome measure for the PGx service.

**Table 2:** Results from pre-workshop survey

	Weighting %	Must do	Should do	Could do	Should not do
*Enabler—In principle receptiveness to pharmacogenomic testing*					
Question—To provide positive feedback to pharmacist to demonstrate effective service delivery, what should the outcome goal of the pharmacogenomic testing service be?					
Number of tests performed	30%	1	1	5	2
% patients satisfied with the service	67%	3	3	3	0
Proportion of patients for whom an intervention is made	85%	5	4	0	0
*Enabler—Appreciation of the benefits of pharmacogenomic testing*					
Question—Pharmacists told us that patients’ may be worried if they find out that their medicines may have been inappropriate, and they need guidance on how to handle this. How should we address this?					
Written information on how to manage this	93%	7	2	0	0
Video of GP explaining how they would manage it	63%	4	2	2	1
Video of patients stating what they would want to be told and how	59%	2	3	4	0
*Enabler—Appreciation of the benefits of pharmacogenomic testing*					
Question—Pharmacists told us that they would like to know the pros and cons of the service to encourage them to take part. How could we best communicate these?					
Pharmacist given a list of Pros and Cons of offering service to the patient	70%	5	2	1	1
Pharmacists given a good quality research paper which outlines pros and cons with evidence provided to back them up	59%	3	3	2	1
Ask pharmacists to list the pros and cons in a workshop working with others	56%	2	2	5	0
*Barrier—Lack of implementation resources to deliver pharmacogenomic testing*					
Question—How do we ensure the whole pharmacy team, peers and line managers are fully engaged prior to and during implementation?					
Set up a prelaunch event to encourage engagement	85%	5	4	0	0
Provide a newsletter outlining the service	63%	1	6	2	0
Provide a free test to all key stakeholders to enable them to understand the service	56%	2	2	5	0
*Barrier—Lack of guidance to design the pharmacogenomic testing service*					
Question—What supporting materials should be produced?					
Introduce SOPs and ask for them to be read and signed by all pharmacy team colleagues	93%	8	0	1	0
Develop patient information leaflets, posters for pharmacy/GP surgeries etc	78%	5	2	2	0
Add online information on company website	67%	1	7	1	0
*Barrier—Lack of knowledge for delivery of a pharmacogenomic testing service*					
Question—What training is required to inform and demonstrate to community pharmacists how to offer and conduct a pharmacogenomic test?					
Written guidance/SOP for pharmacists on how to offer and perform the test	100%	9	0	0	0
Provide an instructional video for pharmacists on how to offer and perform the test	93%	7	2	0	0
Provide a free test for pharmacists to perform on themselves	63%	3	2	4	0
*Barrier—Lack of knowledge for clinical decision making*					
Question—What do community pharmacists (and GPs) need to ensure that they have the knowledge to make safe and appropriate clinical decisions based on the report provided?					
Case studies with answers so they can test themselves	70%	3	4	2	0
Access to a helpline which can help them when decisions are difficult	78%	5	2	2	0
Detailed pharmacogenomic training including underpinning science and how this relates to the information provided	74%	5	1	3	0
*Barrier—Lack of knowledge for clinical decision making*					
Question—What format and frequency should regular summary updates and peer benchmarking information on testing outcomes be?					
Online report	63%	1	6	2	0
Online portal	63%	2	4	3	0
Peer review sessions	48%	1	2	6	0
*Barrier—Interprofessional relationship challenges to deliver pharmacogenomic testing*					
Question—Who should identify the need for the patient to have the test after the prescribing of a new medicine to overcome any inter-professional barriers?					
Community pharmacist	85%	6	2	1	0
General Practitioner (GP)	63%	3	2	4	0
Patient	41%	1	2	5	1

### Workshop

All pharmacist, GPs and patient (n = 12) participants who were invited attended the panel meeting (Table 3) and a good mix of diverse characteristics was noted.

**Table 3:** Characteristics of panel members

Code	Group	Gender	Role	Years in practice	Age Group	Region of England	Size of practice^a^
Ph 1	Pharmacist	F	Pharmacist manager	0–10	n/a	London	5000–10,000
Ph 2	Pharmacist	F	Pharmacy Superintendent	21–30	n/a	National	n/a
Ph 3	Pharmacist	F	Teacher practitioner	0–10	n/a	East	5000–10,000
Ph 4	Pharmacist	F	Pharmacy manager	0–10	n/a	South West	15,000–20,000
Ph 5	Pharmacist	F	Pharmacy manager	31–40	n/a	South West	5000–10,000
Ph 6	Pharmacist	M	Prescribing pharmacist	0–10	n/a	London	5000–10,000
GP 1	Prescriber	M	General practitioner	21–30	n/a	South East	10,000–15,000
GP 2	Prescriber	M	General practitioner	0–10	n/a	South West	15,000–20,000
GP 3	Prescriber	F	General practitioner	0–10	n/a	North East	15,000–20,000
P 1	Patient	F	Patient	n/a	40–60	South East	n/a
P 2	Patient	F	Patient	n/a	> 60	South West	n/a
P 3	Patient	M	Patient	n/a	40–60	London	n/a

^a^Size of practice: prescribers = patient list size, Pharmacists = monthly medicines dispensed

A summary of the discussion for each BCT is presented and discussed with illustrative quotations using the following codes: GP participant = GP, Pharmacist participant = Ph, Patient participant = P. The corresponding number relates to the panel members’ unique code in the consensus panel that they participated in.

### Review outcome goal(s)

Both healthcare professionals valued quality measures over quantity. They also noted the importance of patient satisfaction and the benefit of a confirmatory PGx test result where the patients’ medication was currently suitable and required no amendments. It was also noted that the request for quality measures alone could be a lazy argument with the need for pharmacists to both engage with the service and deliver an agreed minimum quantity of tests in each setting following the investment of setting up the service.

“A league table with numbers of tests wouldn’t be the best way of doing it, but I think there should be some sort of minimum expectation, at least some sort of agreed minimum should be agreed. Because, if you’re doing the work of setting up a service and promoting it, there needs to be some sort of expectation” Ph6.

### Information about emotional consequences

As part of an engagement event, it was felt that the benefits of PGx testing could be enhanced by undertaking a role clarification exercise between the healthcare professionals. GPs reassured pharmacists that they would see an amendment to the prescription as a positive intervention and in no way would it undermine the GP—patient relationship, and that they would support pharmacists by communicating this view to patients. It was however acknowledged by both healthcare professionals that a change of medicine should not be seen as an urgent action, rather one that should be managed in a controlled manner with patients’ expectations being controlled accordingly.

“It’s not that the prescribing has been wrong, or been bad, but that the prescribing was regular, but we can make it better. So that-that we can personalise and enhance the prescribing for you with this test, to improve upon the baseline. That doesn’t have to be the pejorative terms, it’s not wrong prescribing or anything. It's important for the pharmacists to remember is that it’s the prescriber that’s liable” GP1.

### Pros and cons

As part of the training and the onboarding, written information on the pros and cons of offering and delivering the service was seen as beneficial. This included clinical benefits and any patient concerns relating to the use of DNA and the evolving field of genomic medicine.

“They’re going to read all sorts of stuff on the internet and the pharmacists probably need to be armed with a way of dispelling genetic (myths)” P1.

### Social support (practical)

A face to face engagement event was considered desirable and should be attended by pharmacists, their support team (pharmacy technicians and pharmacy assistants) and GPs in a local setting. During the event it was expressed that there should be an opportunity for some panel members to undertake the test on each other for experiential learning. To maintain momentum, this engagement event should be followed up by ongoing and regular communication.

“In the face-to-face event, you could have each of the pharmacists doing the service to each other, and then actually giving each other tests, so you’re combining both in one” Ph6.

### Adding objects to the environment

Development of Standard Operating Procedures (SOPs) for healthcare professionals and information leaflets and short videos on the simplicity and details of the service for patients to view in the pharmacy, GP surgeries and online were all requested as resources to support the implementation.

“I liked that idea, as a patient, being shown maybe in the pharmacies and in the doctor’s surgery, what you have to do. Because I know I know it’s just a swab, but a lot of people might not know that and if it’s just a simple thing, I think that would put a lot of people’s apprehension at rest, if they could see it was just a straightforward thing like that” P1.

### Instruction on how to perform and demonstrate behaviour

The required training for pharmacists to offer and deliver a PGx test includes written guidance and SOPs with a supporting short instructional video. The training should also form part of the engagement event and include content on having difficult conversations with both prescribers and patients.

“It’s not just looking at the practical training or delivering the service, it’s also giving them tools to learn to have difficult conversations with GPs, with patients, etc. because I can imagine some pharmacists will end up having to have difficult conversations” P2.

A unique approach suggested was practical education and training on how to offer the service, how to interpret the reports and how to have positive interactions with prescribers to discuss the potential changes to the patients’ prescription in a community pharmacy setting.

### Feedback on behaviour

Upon reflection, regular summary updates with peer benchmarking were not seen as a high priority; instead, participants requested the use of case studies to practise interpreting patients’ results in a safe learning environment. Furthermore, regular meetings to review service outcomes and discuss interesting case studies were identified as being necessary to provide positive feedback and increase confidence for both healthcare professionals.

“Summary feedback about all the tests that have been done across the town loses any granular detail that you get and unless there’s a major theme of something that keeps going wrong everywhere, or something that’s done really well everywhere, you don’t get much out of it. It might be better to have feedback about a case that you’ve intervened in where the results have been very good, where the patient’s happy and there’s a recognised benefit, so it’s almost if you do ten of these and one of them goes really well, you might learn more from that than the summary of 100 other tests” GP1.

To ensure pharmacists have the knowledge for appropriate clinical decision making, training should be illustrated by case studies and supported with clinical guidelines and flowcharts. Following implementation, a helpline or access to “expert” colleagues was also seen as beneficial by both pharmacist and GP panel members.

“Having a designated person who is an expert that we can contact would be useful. Having to call someone can be a bit of a nuisance. But if you could send them a message and they get back to you with advice, then that would be helpful” GP3.

Regular meetings to discuss clinical outcomes were also requested by pharmacist panel members.

### Credible source

The GP engagement pack should include case studies on the PGx service and include potential savings that could be delivered on prescribing budgets. In addition, GPs requested details of potential benefits for patients and information of the accuracy of the PGx test which were both referenced and evidence-based. Additionally, they requested guidance on how to interpret the test results supported by a clear patient pathway illustrating how the service works and where the GP and pharmacist respectively participate. They were however confident that over time, their confidence and competence to support the pharmacist with amendments to patients’ prescriptions would become part of their daily routine.

“As we roll on and move on, we’ll become more experienced with the genetics and be able to whittle that down to shorter consults” GP3.

Identification of patients suitable for testing should be undertaken by both pharmacists and prescribers for patients on multiple medicines; patients however should not be encouraged to self-refer.

“If it’s ongoing, both could initiate it because patients might say to the pharmacist, “Oh, this is not working,” and so it could be flagged up from either side” GP2.

For newly prescribed medicines the pharmacist could sensitively intervene following delivery of the New Medicines Service (NMS). In the future, with the developing role of the Primary Care Network (PCN), the PCN and practice based pharmacists could play an increasing role in the identification of target patients.

A summary of the agreed strategies for inclusion in the service specification is presented in Table 4.

**Table 4:** Summary of agreed strategies for inclusion in service specification

Barrier/enabler	BCT	Agreed strategy
In principle receptiveness	1.7 Review outcome goal(s)	Use quality outcome measures and agree a minimum number of tests to be delivered
Appreciation of the benefits	5.6 Information about emotional consequences	Initial engagement event to include role clarification with all healthcare professionals
	9.2 Pros and Cons	Detailed benefits of PGx testing and perceived patient concerns for pharmacists to be provided
Lack of implementation resources	3.2 Social support (Practical)	Engagement event to include PGx testing for participants. Provide regular ongoing communication post launch
Lack of guidance to design the service	12.5. Adding objects to the environment	Develop SOPs, information leaflets and short videos to support implementation
Lack of knowledge for service delivery	4.1. Instruction on how to perform behaviour	Engagement event to include training on how to offer the service, supported by written guidance and short instructional video. Include role play of "difficult conversations" between pharmacists, GPs and patients
	6.1 Demonstration of the behaviour	
Lack of knowledge for clinical decision making	2.2. Feedback on behaviour	Use case studies and clinical guidance flowcharts to reinforce underpinning science
Inter-professional relationship challenges	9.1 Credible source	GP engagement pack to include case studies, accuracy of test, potential cost savings, benefits for patients, and patient clinical pathway
		Pharmacists and GPs to identify suitable patients for testing; patients should not self-refer

### Service specification

**Fig. 1:**
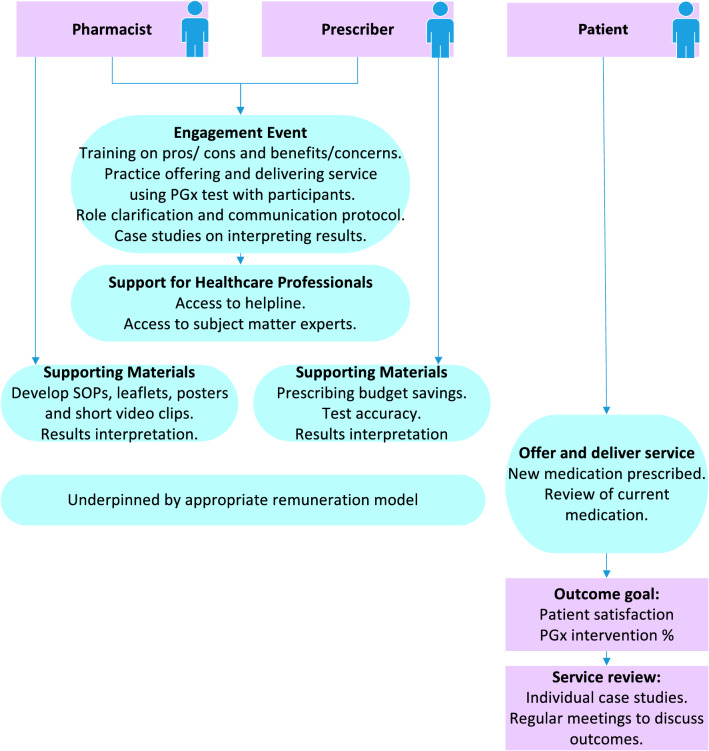
Flow diagram for service delivery

Following feedback from the panel members in the consensus group, the final service specification was developed. As part of the co-design process, the researchers incorporated all the feedback from the pre workshop survey and the comments and the agreed strategies from both the professional and lay panel members in the workshop. A summary flow diagram for the service delivery is presented in Fig. 1.

## Discussion

The online consensus panel of mixed stakeholders reviewed and created a draft service specification for a PGx testing service in a community pharmacy setting in England, which now requires feasibility testing practicability, acceptability and suitability for research purposes. In setting up such a service, a need to address inter-professional relationships was identified as required at the outset, to ensure that different roles overlap, the patient had clarity as to who was responsible for what and that the actions of one healthcare professional were not seen to undermine or contradict the actions of another.

Case based training was identified as being needed by both prescribers and pharmacists to increase their confidence in responding to recommendations with test results. Furthermore, this should be backed up by a helpline to support complex clinical decision-making. Community pharmacists identified the need for some relatively inexpensive support materials to be created, whilst prescribers required evidence of consequences i.e. prescribing budget and test accuracy.

The contribution from the mixed stakeholder group was notable not only for the contributions from their own professional or lay perspective, but also for a clear recognition of how their actions and behaviours were interdependent. Furthermore, the use of a mixed participant consensus panel has identified a novel approach to offering and delivering a PGx testing service in England, and many of these process suggestions could be applied to other community pharmacist led services prior to them being designed, developed, commissioned and implemented.

The results however represented a relatively small number of pharmacists, patients and GPs who by offering to participate may already be demonstrating a positive attitude towards the new technology. Furthermore, all the panel members were purposively sampled and may not be representative of their professional or lay groupings and the imbalance of participants, with six pharmacists and three GPs and patients may have led to a bias in the pharmacists point of view. Additionally, due to the recent pandemic, the consensus panel was held online, and a face-to-face meeting may have generated greater depth in the data. Finally, none of the panel members had any experience of PGx testing and none had any significant understanding of genetic medicine or PGx; their knowledge was limited to their individual academic experiences of introducing and delivering new services.

The study was located in England and included only practitioners from this country, Consequently the service specification and strategies for operationalisation were considered within this context. Some of the required intervention components identified have however been reported previously in other settings e.g. access to appropriate clinical resources for healthcare professionals and patient friendly resources [6]. The need for multi-professional working for effective service delivery is well recognised within the international literature [18] but clearly highlighted here and is likely to be required in any international setting for the reasons identified. Similarly the provision of support for clinical decision making for complex patients is likely to be a ubiquitous requirement. Whilst materials for patients and standard operating procedures need to be designed for local context, the structure and nature of content is likely to be similar across countries.

Both healthcare professions endorsed an engagement event prior to commencement of the service; whilst this is not a common practice currently, and may be a challenge to organise, there was strong support for it to be included. It was recommended that this should be a small local event to include training on the pros and cons of the service, benefits and concerns that patients might perceive and to practise offering and delivering the service using an actual PGx test with healthcare professionals to experience the patient pathway for testing. In addition, a session on role clarification and the communication protocol between pharmacists and prescribers was encouraged; practically an ideal operating model could be developed and discussed at the engagement event. It could then subsequently be adapted for the needs of the healthcare professionals according to the infrastructure of their local system and reviewed and updated post launch if appropriate.

It is interesting to note that when requesting training the participants sought practical guidance i.e. how to perform the test and how to respond to the test results. Education and training for PGx is frequently focused on understanding the underpinning science, which, whilst it may be nice to know, was not seen as a priority.

A request for the availability of a remote subject matter expert or a telephone helpline has not previously been identified in the literature. Given the increasing levels of polypharmacy and subsequent associated complexity, the need for a helpline is intuitive where services are being introduced. Whilst GPs in the UK can access regional medicines information services, the teams within them may want to consider preparing for such PGx clinical decision making enquiries in the future.

The request for Standard Operating Procedures (SOPs) for both pre-emptive and medication review purposes, represents a desire to standardise service delivery and the patient experience. Whilst ubiquitously used in community pharmacies in the UK, there may be different documents or processes used for the same purpose in other countries.

Whilst patient information leaflets for PGx services are likely to be available from companies providing the service, these should ideally be co-designed with patients and carers to ensure appropriateness of content and that most questions and concerns can be adequately answered by this resource.

Interestingly, before engaging with the service, the medical practitioners wanted evidence of the consequences of the testing service i.e. the impact on their prescribing budget and overall test accuracy. Whilst impact on budget may only be relevant in those countries where they are responsible for their budgets, evidence of accuracy is likely to be required to improve confidence in making recommendations. All of these should be relatively inexpensive to address and could ideally be created at a national level.

In terms of how the service should be funded, a mix of productivity, effectiveness, volume and satisfaction was agreed. Traditionally, volume targets have been set for the “*delivery of community pharmacy services in England, irrespective of the size of the pharmacy, volume of dispensing, demographics of the local community or experience or professional capacity of the pharmacist*” [11]. Whilst such a remuneration model results in efficient service delivery, this can sometimes be at the cost of quality and therefore a model which pays for delivery whilst encouraging quality was preferred [19]. The inclusion of general practitioners in the workshop may account for this, as their remuneration model incentivises both delivery and outcomes. The creation of a different remuneration model for a PGx service would therefore require careful consideration in the UK, given the misalignment between the community pharmacy and general practice contracts within the national health system.

## Conclusion

With an underpinning theoretical framework, this study has used a consensus panel of mixed panel members to identify the operationalisation strategies for the previously identified behaviour change techniques for a service specification for delivery of a community pharmacy based PGx service in England. All of the critical feedback and suggestions have been incorporated into a final service specification.

The next steps would be to test this service model on a small scale to ensure that it is acceptable and practical to stakeholders. Feedback should then be sought on all elements to enhance its effectiveness. With no evidence for effectiveness, cost-effectiveness or safety of a pre-emptive PGx testing service model of this nature, a feasibility study testing the service specification outlined here is required before progressing to a definitive trial, if appropriate. Such evidence would then enable the government to decide whether the service provides appropriate value to patients and the health system to enable it to be funded.
